# Development of a Collaborative Education Program for Home Care Nursing Educators’ on-the-Job Training in End-of-Life Home Care: Protocol for a Randomized Controlled Trial

**DOI:** 10.2196/84330

**Published:** 2026-03-27

**Authors:** Ryousuke Yamada, Hanako Numata, Yuka Sumikawa, Kosuke Kashiwabara, Maiko Noguchi-Watanabe, Kayoko Kurita, Noriko Yamamoto-Mitani

**Affiliations:** 1 Department of Gerontological Home Care and Long-Term Care Nursing, Graduate School of Medicine, The University of Tokyo, 7-3-1 Hongo, Bunkyo-Ku, Tokyo, 113-0033, Japan, 81 3-5841-3508; 2 Data Science Office, Clinical Research Promotion Center, The University of Tokyo Hospital, Tokyo, Japan; 3 Center for Research and Development in Higher Education, The University of Tokyo, Tokyo, Japan

**Keywords:** continuing nursing education, end-of-life care, home health nursing, terminal care, on-the-job training

## Abstract

**Background:**

Despite the growing demand for end-of-life home care, nursing educators responsible for on-the-job training face substantial barriers, such as time and geographical constraints, which limit their access to professional development. Collaborative online training programs offer a potential solution to these challenges.

**Objective:**

This study aims to develop and evaluate a collaborative online training program designed to improve the educational ability of home care nursing educators in delivering effective on-the-job training for home-based end-of-life care.

**Methods:**

This parallel group, waitlist-controlled trial recruited home care nursing managers or educators from agencies with at least one home death client in the past year. We will provide this program on the internet. The intervention will comprise three online sessions, each lasting 60 minutes and held monthly, involving one lecture and two interactive case studies related to on-the-job training in end-of-life home care. This will be followed by 8 weeks of weekly educational messages via LINE, a mobile messaging app widely used in Japan. The control group will serve as a waitlist and will receive the same intervention after the control period. The primary outcomes are participants’ attitudes, measured using the End-of-Life Nursing Education Questionnaire. Using a centralized computer-generated randomization system, 125 home care nursing managers and educators will be recruited and randomly assigned to either the intervention or control group at a ratio of 1:1. Stratification will be based on their agency’s annual home death count between November 29, 2023, and November 29, 2024. To minimize performance bias, the intervention content has been standardized before the intervention to ensure fidelity.

**Results:**

This study was approved by the Ethics Committee of the Graduate School of Medicine, The University of Tokyo (2024359NI-(2)) on November 15, 2024. Participant recruitment began on November 29, 2024, and closed on December 25, 2024, with 120 participants enrolled. As of February 15, 2026, the following number of surveys were completed at each time point: 106 at T0 (the intervention group:the control group; 52:54); 103 at T1 (51:52); 102 at T2 (50:52); 40 at T3 and 43 at T4 in the intervention group; and 47 at T5, 44 at T6, and 41 at T7 in the control group. Quantitative data collection was completed by January 2026, and qualitative interviews will be completed by March 2026. Primary quantitative results and integrated results are expected to be submitted for publication in 2026-2027 and 2027-2028, respectively.

**Conclusions:**

This protocol can address geographical and time constraints by providing flexible, online educational resources for home care nursing educators. The anticipated outcomes include enhanced on-the-job training ability among nursing educators for home-based end-of-life care. This study’s findings could support broader implementation of scalable training strategies in home care settings.

## Introduction

With the growing aging population worldwide, the demand for home health care services has increased, as more individuals choose to receive medical care at home while managing chronic illnesses and disabilities [1]. Further, the proportion of individuals desiring to spend their final moments at home has risen, while actual home deaths have also increased. However, providing end-of-life (EOL) care presents several challenges, including physical and psychological distress among clients and families [2].

Home care nursing plays an important role in enabling EOL care at home. Previous studies have identified key factors facilitating home deaths, including medical conditions, personal preferences, and environmental support [3]. Among these, environmental support—including the availability of skilled home care nurses—has been essential for ensuring EOL home care. However, despite an increasing number of home care nursing agencies, a lack of systematic training opportunities for cultivating skilled home care nurses—primarily owing to limited time, funding, and human resources—has been reported as a significant barrier [4]. Therefore, establishing structured training systems to educate skilled nursing staff is crucial.

One of the key factors for enabling EOL home care is the availability of systematic training opportunities within the home care nursing agencies. Training opportunities for home care nurses at their agencies have been shown to contribute to improved staff retention and ability [5,6]. Several external training programs about EOL care, such as the End-of-Life Nursing Education Consortium [7] and the Program of End-of-Life Care for Home Visiting Nurses Training [8], provide foundational EOL care knowledge. However, as EOL home care requires the practical application of this knowledge within individual clients [9], such foundational knowledge is insufficient. On-the-job training (OJT) is particularly important in home care nursing agencies [10] because it can be provided in a tailored manner to suit the individual needs of each staff member and the client’s context. In addition, among these constraints on time, funding, and human resources that challenge systematic training, OJT offers a practical option by being integrated into daily work. Nevertheless, home care nursing managers and staff have reported unsatisfactory preparation for delivering effective EOL home care [11].

Home care nursing managers and educators face challenges in attending external training programs owing to geographic and time constraints. Particularly, rural home care nursing agencies have limited access to such programs [12]. Furthermore, almost all home care nursing agencies have only one designated home care nursing manager. This single manager is responsible for multiple roles, including that of an educator [13]. This is why they often lack the time and scheduling flexibility to participate in lengthy in-person training courses, which require several hours to multiple days. Although some home care nursing agencies in Japan have staff designated as educators [14], most employ only one person in this role. Therefore, both managers and educators often face similar barriers in accessing training opportunities. Based on these limitations, a practical and accessible program that specifically supports educators in delivering OJT in EOL home care is needed.

To address these challenges, we will develop and evaluate an online training program designed specifically for home care nursing managers and educators to improve their educational abilities in conducting OJT for EOL home care. Given the geographic and time constraints of these managers and educators, we will develop a program that is entirely online, using a private messaging app for interaction to provide a short, flexible, and accessible learning format.

This study will evaluate the effectiveness of this training program through a randomized controlled trial (RCT). The primary outcome will be fostering changes in participants’ attitudes as educators. Secondary outcomes will include improvements in knowledge and educational practices related to OJT in EOL home care. This study aims to establish a scalable evidence-based training model for widespread implementation in home care nursing agencies, thereby enhancing the quality of EOL home care.

## Methods

### Ethical Considerations

This study was approved by the Ethics Committee of the School of Medicine, The University of Tokyo (2024359NI-(2)). We will use an informed consent form to explain the study’s purpose, voluntary participation, and privacy protection to all participants before enrollment. This is a minimal risk study. If participants experience distress or feel unwell, they can choose to pause or discontinue participation at any time. The principal investigator will serve as the contact point for any concerns. We will use an informed consent form to explain the study’s purpose, voluntary participation, and privacy protection to the participants. After obtaining the consent of all participants, the study procedures will be conducted in accordance with the ethical guidelines of The Medical and Health Research Involving Human Participants in Japan [15] and the 1964 Declaration of Helsinki. Regarding incentives, participants will receive ¥5000 upon completion of all required surveys through T4 (course A) or T7 (course B; an exchange rate of ¥159.49 to US $1 is applicable). An additional ¥1000 will be provided to participants who contribute real-world educational cases for the case study sessions and to those who participate in interviews. Participants and staff members who participate in interviews will receive ¥1000 immediately after interview completion. No payments will be made to participants who withdraw before completing the required procedures. No additional compensation for harm is planned.

### Design

This study will be an RCT whose design, implementation, and reporting adhere to the SPIRIT (Standard Protocol Items: Recommendations for Interventional Trials) guidelines [16]. This RCT has been registered with the UMIN Clinical Trials Registry (UMIN000056165).

### Setting

The program will be conducted via Zoom (Zoom Communications), a video communication platform. The sessions will include lectures, case studies, and interactive communications to enhance participants’ learning and engagement. LINE, a freeware app for instant messaging and social networking developed by LY Corporation, will be used to distribute weekly educational messages and facilitate communication among participants throughout the intervention period.

### Eligibility Criteria for Participants

The eligibility criteria for participants are as follows: home care nursing managers or educators (staff primarily responsible for education in their agency) working at an agency with at least one home death client in the previous year; access to Zoom using a computer; the ability to attend all scheduled training program sessions; and fluency in Japanese, which is defined as the capacity to read and understand written materials in Japanese.

### Intervention Program

We developed an educational program for home care nursing managers and educators to improve their educational ability in OJT in EOL home care. The intervention group will receive this program after the baseline survey (T0) on a specific day. The control group will constitute a waitlist lasting 4 months after the baseline survey, and the same program will be delivered after the third survey (T2).

### Program Theory of This Project

**Figure 1.**
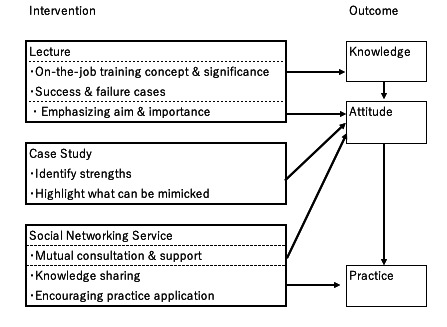
Conceptual framework of the training program.

This section outlines the theoretical framework that guides the design and structure of our educational program. This project is primarily based on the theory of transfer of learning [17], which explains how knowledge is applied in the workplace post training. To apply this theory, our program’s design focuses on three key domains: (1) trainee characteristics, (2) training design, and (3) work environment. The conceptual framework illustrating how these intervention components are designed to influence outcomes is presented in Figure 1.

First, to address trainee characteristics, the model emphasizes the importance of high motivation, confidence, and positive outcome expectations to ensure effective training. To achieve this, we emphasize the aim and significance of the program based on the ARCS (attention, relevance, confidence, and satisfaction) model [18]. For example, at the beginning of the content, we present the objectives and goals that directly relate to real-world challenges faced by participants, thereby capturing their attention and establishing relevance. Second, regarding training designs, this program is grounded in Merrill’s First Principles of Instruction [19] to ensure the learning is practical and effective. This program activates the participants’ prior experience and facilitates the learning of new information in the lecture session. Subsequently, participants engage in application and integration in the following case study session, where they use both their experiential and new knowledge to solve real-world problems. Third, to support the work environments and promote long-term maintenance of skills, we provide ongoing reinforcement. Based on previous research showing that social networking service–based messaging was an effective tool for prompting action [20], we use LINE for this purpose. Through this platform, we send weekly messages to reinforce key concepts from the sessions, prompt the application of skills, and foster a peer support community.

### Program Content and Delivery

**Figure 2.**
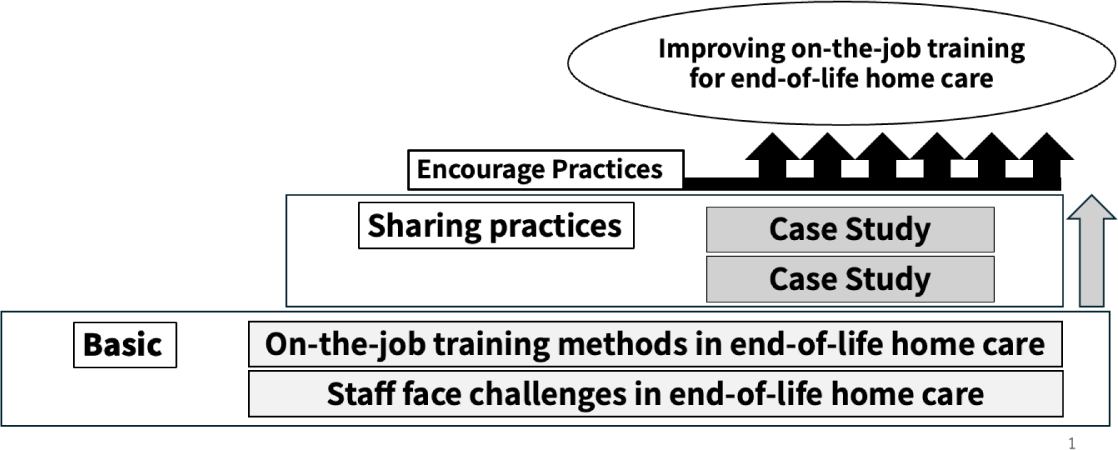
Graphic syllabus of this program.

The program consists of three sessions: one lecture and two case studies, each lasting 60 minutes and conducted once every third Sunday of the month. This structure is based on the quality improvement collaborative approach, which promotes learning through periodic collaborative meetings supported by intersession communication [21]. This program is composed of three main types of sessions: a lecture, a case study, and weekly messages via LINE, which are designed to build on each other. The detailed objectives, goals, and content for each component are outlined in Textbox 1. The overall objective is for learners to be able to conduct effective OJT with adequate knowledge and attitude in EOL home care. A graphic syllabus provides participants with a clear, visual road map of this learning process (Figure 2).

Textbox 1. **Table of contents.**

**Course objective**

Learners can conduct effective on-the-job training with adequate knowledge and attitude for staff providing end-of-life home care.

**Course goals**

1. Learners will state adequate knowledge about on-the-job training in end-of-life home care.
2. Learners will describe practical educational methods that can be implemented in practice.
3. Learners will build confidence and motivation to conduct on-the-job training in end-of-life home care.
4. Learners will clarify their uncertainties regarding educational methods.

**Program**

**Lecture objective**

Learners can acquire educational knowledge and attitudes to conduct on-the-job training in end-of-life home care.

**Lecture goals**

1. Learners will identify challenges staff encounter during end-of-life home care.
2. Learners will describe the educational support required to address these challenges.

**Lecture contents**

1. The current situations and challenges faced by staff providing end-of-life home care.
2. Concrete methods for conducting effective on-the-job training in end-of-life home care.

**Case study objective**

Learners can understand practical educational methods in on-the-job training for staff providing end-of-life home care.

**Case study goals**

1. Learners will explain practical educational methods for on-the-job training in end-of-life home care.
2. Learners will gain confidence and motivation to implement on-the-job training in their practice.
3. Learners will be able to apply the acquired methods to conduct effective on-the-job training starting immediately.

**Case study contents**

1. Learners will understand one real-world case shared by a representative.
2. Group discussions will focus on best practices for conducting on-the-job training in end-of-life home care, based on a case scenario.
3. Learners will connect the knowledge from a lecture to a case scenario, fostering reflective and collaborative learning.

Session 1 is a lecture on foundational principles. We introduce educational methods for staff involved in EOL care, structured within a framework that is grounded in previous research findings and educational theory. Moreover, we highlight the common challenges that staff encounter in providing EOL care. The session concludes with a short group activity based on a real case designed to encourage practical reflection and discussion. Sessions 2 and 3 depict case studies. Providing tailored OJT in EOL home care requires applying practical knowledge to various situations; for this reason, these sessions focus on practical application. Discussing these real-world cases helps participants learn from diverse experiences and acquire the practical knowledge necessary for their practice. To ensure relevance, a real-world case based on their actual educational experiences is gathered beforehand from volunteering participants through an online interview. The case details focus on the educational interactions between the educator, staff, and the client. After gathering a case, we summarize it in a table to facilitate discussion. The content is anonymized, and case details not essential for educational discussion are omitted. The case materials include no identifiable client information; accordingly, client consent was not required, as confirmed by the ethics committee. The anonymized cases are reviewed by five nursing researchers, including an expert in home care nursing and case study methodology, to ensure educational relevance and validity. In the sessions, participants analyze the case scenario, discuss best practices as educators, and connect theoretical knowledge to the practical situation, fostering reflective and collaborative learning. The goal is for participants to improve attitudes and be able to apply the acquired methods in their own practice.

To provide continuous support and promote the application of learning, a LINE group is used between sessions. The group, created immediately after the lecture, serves two main functions. The primary function is to prompt action and reinforce key concepts through regular messages sent by the researchers. These messages start after the first case study and continue weekly at 6 PM on Fridays for 8 weeks. We post brief messages weekly to reinforce key learning points from the program and encourage reflection and peer discussion about OJT for EOL home care. The secondary function is to provide a community for peer support. This platform allows participants to share experiences and mitigate the professional isolation common among educators. To ensure sustainable support, the community remains accessible for participants’ use even after the study concludes. Participation in the LINE group is voluntary. Participants may opt out of the online messaging component at any time by leaving the LINE group, and this will not affect their participation in the study or completion of the surveys. Participants may interact by reading messages, posting text or images, replying to posts, and using reactions; passive participation (reading only) is also permitted. If inappropriate content is posted, the research team will request deletion. Messaging data used for research will be deidentified before analysis.

### Measures

All measurements will be taken at the individual level using a self-administered survey. T0 is the baseline survey before the program begins. T1 is administered immediately after the lecture (session 1), and T2 immediately after completion of the full program. For the early intervention group, follow-up surveys are administered 3 and 6 months after T2 (T3 and T4, respectively). For the waitlist control group, surveys are administered at the same schedule during the waiting period (T0 to T2) and then immediately after they complete the program and at 3 and 6 months post program (T5, T6, and T7, respectively). Depending on group assignment (early intervention group: T0, T1, T2, T3, T4; waitlist control group: T0, T1, T2, T5, T6, T7), all variables, except covariates, will be measured at 5 or 6 time points. Unless otherwise indicated, the survey items use a 5-point Likert scale, wherein one represents “strongly disagree” and five represents “strongly agree.”

### Primary Outcome

The primary outcome is participants’ attitudes as educators in EOL care, assessed using the End-of-Life Nursing Education Questionnaire (ELNEQ). The ELNEQ consists of five domains: confidence in teaching, motivation for teaching, preparation to provide teaching, preparation to lead initiatives in EOL care, and expected influences on participants. For this study, we selected three domains as the primary focus: confidence in teaching, motivation for teaching, and expected influences on participants. These domains were chosen because attitudinal changes are key predictors of behavioral change in practice [22]. Higher scores on the ELNEQ indicate more positive attitudes toward EOL care education. The ELNEQ consists of 12 items evaluated on a 5-point Likert scale, where one represents “strongly disagree” and five represents “strongly agree.” Sample items include “I am satisfied with my ability to teach EOL care” and “I am motivated to teach EOL care.” The reliability of these domains was demonstrated in the original study [23], with Cronbach α values of 0.86 (confidence in teaching), 0.94 (motivation for teaching), and 0.93 (expected influences on participants). The effect sizes of Cohen *d* in the development study of confidence in teaching, motivation for teaching, and expected influences on participants are 0.75, 0.13, and 0.58, respectively [23]. To calculate the sample size, given the feasibility of the study and the expectation that participants will be highly motivated by education, an effect size of 0.58 was used. Finally, the ELNEQ’s psychometric properties, including face validity, content validity, construct validity, and reliability, have been established in a prior study, supporting its suitability for this research. In this study, the primary end point is the change in this outcome from baseline (T0) to post intervention (T2).

### Secondary Outcomes

The first secondary outcome is the participants’ knowledge about the roles and responsibilities of educators in EOL home care. This knowledge encompasses understanding required care practices, the systems and roles of educators, and OJT methods for staff providing EOL home care. A scale was developed based on Shulman’s pedagogical content knowledge theory [24]. The theory includes six categories: content knowledge; curriculum knowledge; pedagogical content knowledge; knowledge of learners and their characteristics; knowledge of educational contexts; and knowledge of educational ends, purposes, and values and their philosophical and historical grounds. These categories were adapted to fit the context of EOL home care nursing. The scale consists of six items, evaluated on a 5-point Likert scale, wherein one represents “strongly disagree” and five represents “strongly agree.” Sample items include “I have adequate knowledge about required care practices at end-of-life home care as an educator” and “I can clearly describe the system and role of educator for OJT at end-of-life home care.” A higher score implies a higher level of knowledge about educators in EOL home care.

The second secondary outcome is the attitude of educators in EOL home care. This scale was developed based on Bandura’s theory and previous research [22,25], and assesses confidence, motivation, and outcome expectations. It consists of three items, measured on a 5-point Likert scale, wherein one represents “strongly disagree” and five represents “strongly agree.” It includes three items: “I am confident in my ability to teach staff about on-the-job training in end-of-life home care”; “I will actively teach staff who provide end-of-life home care”; and “I believe my education can positively influence staff, enabling them to gain confidence in providing end-of-life home care.” A higher score implies a more positive attitude among educators in EOL home care.

The third secondary outcome is the practice of educators in EOL home care. Key components of practice were extracted from a previous study and converted into a measurable scale [26], which includes five items, evaluated on a 5-point Likert scale, wherein one represents “never” and five represents “always.” It includes five items, such as “I confirm whether staff have sufficient readiness to provide end-of-life home care” and “I assign clients to staff based on their experience and skill level.” A higher score implies more frequent educational practice in EOL home care.

Finally, we will evaluate general educational practice based on Oiwa et al’s [27] research. From this study’s four domains, we selected factor 3 (attitude and behavioral skills as an educator). This scale includes five items, such as “I create an open environment where learners feel comfortable consulting me” and ”I interact with learners in a respectful manner.” A higher score implies better overall practice as an educator in a general hospital context.

### Covariate

Demographic attributes, including gender, age, and participants’ educational background, will be collected in EOL care. Additionally, workplace-related information will be gathered, such as agency size, the number of home deaths managed annually, and whether the agency receives additional payment, such as 24-hour home care nursing support or high-function home care nursing. In Japan, high-function home care nursing is one of the additional payments that can be obtained by agencies that meet numerous requirements, such as the number of deaths in their homes, pediatric clients, and so on.

### Participants and Procedure

#### Recruitment and Randomization

**Figure 3.**
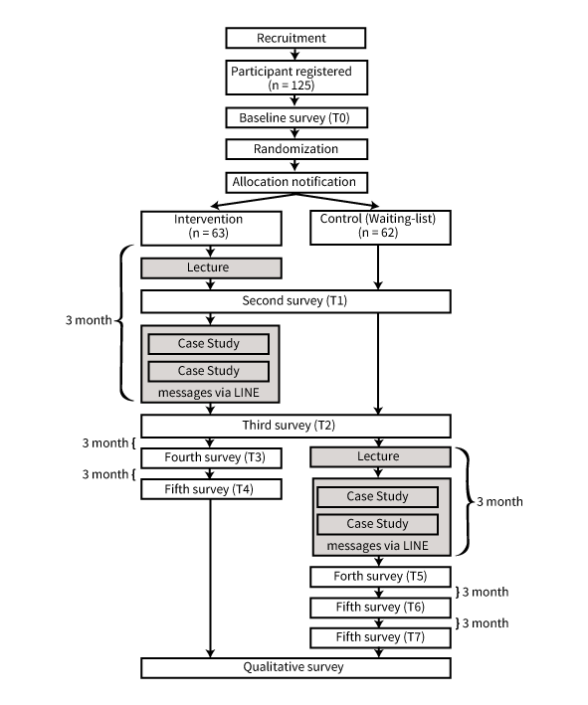
The study protocol flowchart shows participant recruitment, allocation, and intervention timeline. T0 represents the baseline survey; T1 is administered immediately after the lecture (session 1); T2 is administered immediately after completion of the full program. Follow-up surveys are conducted at 3 and 6 months after T2 for the intervention group (T3 and T4, respectively) and at corresponding intervals after the waitlist control group completes the program (T5 to T7).

Figure 3 illustrates the flow of participant recruitment and selection in this study. We aim to recruit 125 home care nursing managers and educators. Recruitment will be carried out through online platforms such as NsPace (a community space for home care nurses managed by Teijin, Japan) and our university’s laboratory website. Further, snowball sampling will be used by asking participants to share study information with their colleagues who meet the inclusion criteria. Information on this study will include the objectives, procedures, and registration methods.

To allocate participants to the intervention and control groups at a 1:1 ratio, restricted randomization with stratification will be performed. Participants will be stratified into three groups based on the number of home deaths managed by their agency between November 29, 2023, and November 29, 2024, as reported at T0: fewer than 5, 5 to 10, and more than 10, following the criteria of previous studies [28]. Participants will be randomized to either course A (earlier program) or course B (later program) using the UMIN Internet Data and Information System for Clinical and Epidemiological Research (Cloud version). The allocation sequence is automatically generated by a centralized system using permuted block randomization with variable block sizes, ensuring appropriate allocation concealment. The lead investigator will assess participant eligibility, enroll them, and perform the allocation within the system. Allocation concealment is strictly maintained; the assigned group is only revealed by the centralized system after the participant is formally registered and assigned. As this is a waitlist-controlled trial, participants will be informed of their program schedule 14 days before the program begins; thus, this study is conducted in an open-label format without participant blinding. To minimize the risk of performance bias, the intervention will be delivered with fidelity according to preplanned content. In addition, to reduce the risk of reporting and expectation biases in the intervention group, we will use validated and standardized scales for our primary outcomes to ensure measurement consistency.

Baseline data (T0) will be collected immediately after registration. Two weeks before the program begins, participants will be informed of their group schedule (course A or course B). Course A participants will attend the first program session, which consists of a lecture delivered via Zoom. Immediately after the lecture, a follow-up survey (T1) will be administered to all participants. Following the lecture, course A participants will be added to a LINE group and registered with our official LINE account. One month later, a case study session will be held. Additionally, weekly educational messages will be sent every Friday at 6 PM for 8 weeks (a total of 8 messages). After completing all interventions, the third survey (T2) will be conducted immediately after the intervention period. Subsequently, the fourth survey (T3) will be conducted 3 months after T2, while the fifth survey (T4) will be conducted 3 months after T3. Course B participants will continue their routine clinical practice from T0 to T2 and will then participate monthly in the program (the lecture and two case studies). Surveys T5, T6, and T7 will be conducted at corresponding intervals after course B completes the program.

Following quantitative evaluations, a qualitative assessment will be conducted. At T4 (course A) or T7 (course B), participants will be asked whether they can join an interview alongside their staff. We plan to interview approximately 20 educators and 20 staff members. Participants will be recruited by convenience sampling (volunteers). For participants, questions will focus on changes in OJT after the training program and perceived variations in staff performance. The questions from the staff will address the education received from the educators. Interviews will be conducted online via Zoom, last approximately 60 minutes, and will be audio-recorded. To protect confidentiality and encourage feedback, interviews for educators and staff will be conducted in entirely separate sessions. Transcripts will be prepared by the research team and anonymized after transcription. Data will be analyzed using thematic analysis. Initial coding will be conducted by the lead investigator, and emerging codes and themes will be iteratively reviewed and discussed by the research team to enhance analytic rigor and credibility.

#### Sample Size

The required sample size was calculated to detect participants’ change in the primary outcome, specifically participants’ attitudes as educators. The required sample size (n=96) was calculated using G*Power (version 3.1; Universität Düsseldorf), assuming a power of 80%, an α level of .05, and an effect size *d* of 0.58. To account for a projected dropout rate of 25%, we determined a final target sample size total of 125 participants.

### Data Management and Analysis

All quantitative data will be collected using SurveyMonkey. Data will be downloaded by the lead investigator and stored on a password-protected file within a locked cabinet at the research institution. Identifiable source data (eg, data linkage key files and unencrypted recordings) will be accessible only to the lead investigator; deidentified, analysis-ready datasets and anonymized transcripts will be shared with the research team only after all personal identifiers have been removed. Related to the qualitative data governance, to strictly maintain participant privacy, interview recordings and transcripts for educators and staff will be stored in separate secure digital folders. Regarding digital platform security, Zoom sessions (lecture and case studies) will be recorded and chat logs saved for fidelity assessment; recordings will also be shared with participants who were absent from a session. Zoom data will be stored in the Zoom cloud, with access limited to the lead investigator. LINE group posts (text, images, and stickers) will be analyzed after anonymization; engagement will be summarized as counts of reads and reactions, and will be collected by manual counting rather than export. All study data will be retained for 10 years. As this is a waitlist-controlled trial evaluating an educational program with minimal risk to participants, no formal data monitoring committee was established. The research team, led by the principal investigator, will oversee the trial progress, data integrity, and participant safety. No interim analyses are planned, and no stopping guidelines have been established, given that the intervention carries minimal risk to participants. Any important modifications to the protocol that may impact the conduct of the study, including changes to study objectives, study design, sample sizes, or eligibility criteria, will be reported to the Ethics Committee of the Graduate School of Medicine, The University of Tokyo, and updated in the UMIN Clinical Trials Registry. Statistical analyses will be performed using RStudio (version 2022.12.0+353; Posit PBC). All *P* values will be 2-sided, and a threshold of *P* less than .05 will be considered statistically significant. We will perform an intention-to-treat analysis, including all participants who were randomized as the primary analysis. The primary effect will be estimated using analysis of covariance (ANCOVA) with the outcome at T2 as the dependent variable and group and baseline (T0) as covariates. Missing outcome data will be handled using a complete case approach.

### Estimation of a Lecture Effect

As part of the process evaluation, we will evaluate the effect of lectures on the primary and secondary outcomes of T0 (baseline) and T1 (post lecture). Primary and secondary outcomes, which will be continuously distributed and measured at T0 and T1, will be analyzed using ANCOVA; a linear model will be used where each outcome at T1 is the dependent variable and T0 and group are the covariates. If the assumption of homogeneity of regression slopes is violated, an interaction term between the baseline values and group assignment will be tested.

### Estimation of Overall Intervention Effect

As this study’s primary analysis, we will evaluate the overall intervention effect on the primary and secondary outcomes of T0 (baseline) and T2 (post intervention). Primary and secondary outcomes will be analyzed with ANCOVA using a linear model where each outcome at T2 is the dependent variable and T0 and group are the covariates. If the assumption of homogeneity of regression slopes is violated, an interaction term between the baseline values and group assignment will be tested.

### Estimating the Duration of the Overall Intervention Effect

To evaluate the duration of the treatment effect, primary and secondary outcomes will be assessed at T0 and at three postintervention time points for both groups: T2, T3, and T4 for the intervention group, and T5, T6, and T7 for the control group, representing immediately after, 3 months after, and 6 months after the intervention, respectively. We will use repeated measures ANOVA, with time as the within-subject factor and T0 or T2 (baseline) as a covariate. To determine whether the intervention effect persists, diminishes, or increases over time, mean values will be compared between adjacent time points (eg, T2 to T3 and T3 to T4 for the intervention group, and T5 to T6 and T6 to T7 for the control group).

## Results

The study protocol was approved by the Ethics Committee of the School of Medicine, The University of Tokyo (2024359NI-(2)) on November 15, 2024. Participant recruitment started on November 29, 2024, and closed on December 25, 2024, with 120 participants enrolled. As of February 15, 2026, the following number of surveys have been completed: 106 at T0 (course A:course B; 52:52); 103 at T1 (52:51); 102 at T2 (50:52); 40 at T3 and 43 at T4 for course A; and 43 at T5, 38 at T6, and 41 at T7 for course B. Quantitative data collection was completed by January 2026, and qualitative interviews will be completed by March 2026. These data demonstrate the feasibility of recruitment and follow-up within the planned timeline. Primary quantitative results and integrated findings are expected to be submitted for publication in 2026-2027 and 2027‐2028, respectively.

## Discussion

This program aims to enhance the ability of home care nursing educators in providing OJT in EOL home care through multiple approaches. Further, we will investigate the staff experience with educational practices conducted by their educator. This study’s strength is that it presents a strategy for the dissemination of a required course for home care nursing educators while addressing time and geographic constraints. First, this program mitigates the time constraints of training by conducting a 60-minute session once a month on a Sunday. Moreover, it may be possible for home care nursing educators to take the course in their spare time because home care nursing agencies tend to be closed on Sundays. Second, based on an online training program, we explain how to address geographically challenging situations of home care nursing agencies. Home care nursing agencies in rural areas tend to find it difficult to use off-the-job training. Therefore, providing an online training program may resolve this situation. Additionally, this program will be feasible to deliver and evaluate within this trial. In Japan, there are 15,055 designated home care nursing agencies as of August 2024 [29]. Because home care nursing managers or educators often cover OJT within their agencies, the potential target population could be on the order of one educator per agency (approximately 15,000 nationwide), although this is an estimate. If this trial demonstrates feasibility and effectiveness, the program may support broader dissemination in the future.

In this study, rewards will be provided to reduce the burden on participants. This additional payment may affect changes in outcomes; however, we minimized this risk by providing payment after the survey responses. In addition, all outcome measures in this study are self-reported. Another obstacle is represented by the difficulty in excluding the risk of social desirability, reporting, and expectation biases in the intervention group. To partially address this limitation, we will use validated and standardized scales for our primary outcomes to ensure measurement consistency. In addition, we will conduct interviews with a subset of staff members who received training from the participants 6 months after this program. These staff interviews are intended to supplement the self-reported data by providing external perspectives on behavioral changes.

This RCT will be the first experimental study regarding a training program for educators in EOL home care and will explain a dissemination strategy. If successful, this program can become an evidence-based model that can be used as a framework for educators in other care settings. The diffusion of training programs in the workplace will encourage staff who provide EOL home care to carry out their duties with sufficient confidence in their workplace.

## Abbreviations

ANCOVA: analysis of covariance
ARCS: attention, relevance, confidence, and satisfaction
ELNEQ: End-of-Life Nursing Education Questionnaire
EOL: end-of-life
OJT: on-the-job training
RCT: randomized controlled trial
SPIRIT: Standard Protocol Items: Recommendations for Interventional Trials
